# Does the Urinary Microbiome Play a Role in Urgency Urinary Incontinence and Its Severity?

**DOI:** 10.3389/fcimb.2016.00078

**Published:** 2016-07-27

**Authors:** Lisa Karstens, Mark Asquith, Sean Davin, Patrick Stauffer, Damien Fair, W. Thomas Gregory, James T. Rosenbaum, Shannon K. McWeeney, Rahel Nardos

**Affiliations:** ^1^Division of Bioinformatics and Computational Biology, Oregon Health and Science UniversityPortland, OR, USA; ^2^Division of Urogynecology, Oregon Health and Science UniversityPortland, OR, USA; ^3^Division of Arthritis and Rheumatology, Oregon Health and Science UniversityPortland, OR, USA; ^4^Department of Behavioral Neuroscience, Oregon Health and Science UniversityPortland, OR, USA; ^5^Department of Psychiatry, Oregon Health and Science UniversityPortland, OR, USA; ^6^Advanced Imaging Research Center, Oregon Health and Science UniversityPortland, OR, USA; ^7^Devers Eye Institute, Oregon Health and Science UniversityPortland, OR, USA; ^8^Kaiser PermanenteClackamas, OR, USA

**Keywords:** bladder disease, bladder microbiome, urinary microbiome, overactive bladder, human microbiome, urinary incontinence, urge

## Abstract

**Objectives:** Traditionally, the urinary tract has been thought to be sterile in the absence of a clinically identifiable infection. However, recent evidence suggests that the urinary tract harbors a variety of bacterial species, known collectively as the urinary microbiome, even when clinical cultures are negative. Whether these bacteria promote urinary health or contribute to urinary tract disease remains unknown. Emerging evidence indicates that a shift in the urinary microbiome may play an important role in urgency urinary incontinence (UUI). The goal of this prospective pilot study was to determine how the urinary microbiome is different between women with and without UUI. We also sought to identify if characteristics of the urinary microbiome are associated with UUI severity.

**Methods:** We collected urine from clinically well-characterized women with UUI (*n* = 10) and normal bladder function (*n* = 10) using a transurethral catheter to avoid bacterial contamination from external tissue. To characterize the resident microbial community, we amplified the bacterial 16S rRNA gene by PCR and performed sequencing using Illumina MiSeq. Sequences were processed using the workflow package QIIME. We identified bacteria that had differential relative abundance between UUI and controls using DESeq2 to fit generalized linear models based on the negative binomial distribution. We also identified relationships between the diversity of the urinary microbiome and severity of UUI symptoms with Pearson's correlation coefficient.

**Results:** We successfully extracted and sequenced bacterial DNA from 95% of the urine samples and identified that there is a polymicrobial community in the female bladder in both healthy controls and women with UUI. We found the relative abundance of 14 bacteria significantly differed between control and UUI samples. Furthermore, we established that an increase in UUI symptom severity is associated with a decrease in microbial diversity in women with UUI.

**Conclusions:** Our study provides further characterization of the urinary microbiome in both healthy controls and extensively phenotyped women with UUI. Our results also suggest that the urinary microbiome may play an important role in the pathophysiology of UUI and that the loss of microbial diversity may be associated with clinical severity.

## Introduction

Nearly 19% of women over the age of 44 suffer from Urgency Urinary Incontinence (UUI; Stewart et al., 2003)—a strong sensation of the need to urinate (“urgency”) followed by immediate leakage of a large volume of urine. The social, psychological, and economic burden of this disorder are enormous (Stewart et al., 2003; Norton and Brubaker, 2006; Coyne et al., 2011). Fear of losing urinary control is associated with depression, anxiety, social isolation, reduced functional status, increased risk of institutionalization and overall negative impact on quality of life (Coyne et al., 2013). The economic burden of UUI is projected to be $82.6 billion by 2020 (Coyne et al., 2014).

Despite the devastating impact of UUI, we have yet to fully understand its underlying pathophysiology which is a significant barrier to developing targeted interventions geared toward eliminating or reducing symptoms. UUI is commonly attributed to abnormal neuromuscular signaling causing involuntary bladder muscle contractions known as detrusor overactivity (DO). However, DO is only observed in approximately 58% of women with UUI (Hashim and Abrams, 2006) and treatments aimed at reducing DO are ineffective for approximately half of the people that use them (Nitti et al., 2010). Even in those with DO, it is unclear what underlying factors contribute to this urodynamic observation. These findings suggest that more attention needs to be placed on understanding pathophysiologic mechanisms that contribute to UUI. Understanding these mechanisms is a necessary step toward improving effectiveness of existing therapy and introducing new therapeutic approaches.

Emerging evidence indicates that a shift in the normal microbiome of the bladder may play an important role in pathophysiology of UUI. Currently, the diagnosis of UUI requires exclusion of acute urinary tract infection (UTI), as there is large overlap in symptomatology. Thus, standard of care rules out an infectious etiology as a possible cause for UUI symptoms. However, the long-held belief that urine is sterile in the absence of an acute UTI has been recently challenged. New techniques such as 16S rRNA gene sequencing (Nelson et al., 2010; Siddiqui et al., 2011; Fouts et al., 2012; Wolfe et al., 2012; Lewis et al., 2013) and expanded quantitative urine culture techniques (EQUC; Hilt et al., 2014; Pearce et al., 2014) have revealed that urine samples actually contain a variety of bacteria, many of which are not detectable by standard culturing techniques. The use of 16S rRNA gene sequencing and EQUC techniques have allowed researchers to identify bacteria that are uncultivable under standard culture conditions, and have identified a polymicrobial community of bacteria that reside in the urinary tract.

The role of bacteria in the urinary tract—known as the urinary microbiome—in health and in urogenital disorders is largely unknown. Asymptomatic bacteriuria, the presence of bacteria in a urine specimen from a patient without signs or symptoms of a UTI- occurs in the general population in individuals with no known urinary tract disorder (Colgan et al., 2006). Importantly, there is also evidence suggesting that these bacteria may modulate UUI pathogenesis- at least for a subset of women. Low-count [<10^5^ colony forming units per milliliter (CFU/mL)] bacteria in urine has been associated with symptoms of overactive bladder syndrome (OAB; Sorrentino et al., 2015), including UUI (Brubaker et al., 2014). Further, altered bacterial communities have been identified in women with UUI in studies that have performed EQUC and 16S rRNA gene sequencing (Hilt et al., 2014; Pearce et al., 2014; Thomas-White et al., 2016). These studies provide evidence that OAB symptoms, including UUI, could be influenced by alterations in the urinary microbiome. However, these studies had several characteristics that were different between their UUI and control cohorts, such as age, BMI, and hormonal status, all of which may relevantly affect the urinary microbiota composition. Furthermore, there are only a handful of urinary microbiome studies to date, underscoring the great need for more research in this area.

The goal of this prospective study is to characterize the urinary microbiome in health and in UUI, and to determine whether differences in microbial composition and diversity are observed between these groups. We also wanted to identify possible relationships between symptom severity and the composition of the urinary microbiome in women with UUI. We sampled the urinary microbiota by amplification of the 16S rRNA gene followed by sequencing on the Illumina MiSeq platform. Our participants (cases with UUI and controls with normal bladder function) are well phenotyped for bladder function with detailed validated bladder questionnaires and attention is given to minimize confounding variables such as menopausal and estrogen status. Our results indicate that there is a polymicrobial community in the female bladder in both healthy controls and women with UUI. We identify statistically significant differences in the relative abundance of specific bacteria in women with UUI compared to controls. We also show that lower microbial diversity is associated with increase symptom severity in women with UUI. These results suggest that the urinary microbiome may play an important role in the pathophysiology of UUI and that the loss of diversity may be associated with clinical severity.

## Materials and methods

### Participants and sample collection

This case-control study was conducted at Oregon Health and Science University (OHSU). The study was approved by OHSU's Institutional Review Board (IRB 10729) and written consent was obtained from all participants. Participants were women between the ages of 40 and 70 recruited from the general public through flyers posted around OHSU's Center for Women's Health and public areas around OHSU. We recruited 10 women with normal bladder function (controls), and 10 women with daily UUI (cases). Control subjects included females without any history of overactive bladder syndrome or current urge urinary incontinence, urgency, or frequency. Case subjects included females with daily UUI for at least the previous 3 months. For both groups, symptom qualifications were confirmed by medical history and 3-day bladder diary that included the frequency of urination, leakage episodes (if any), volume of fluid intake, and assessments of urgency through the urgency severity scale (USS; Cartwright et al., 2011). Exclusion criteria included history of frequent stress urinary incontinence (>once a week), prior urinary incontinence surgery, symptomatic pelvic organ prolapse, history of chronic pelvic pain lasting more than 3 months, history of pelvic irradiation or bladder cancer, current urinary tract infection, antibiotic use in the previous month, and past or present neurological diseases such as stroke, epilepsy, multiple sclerosis, traumatic brain injury, and spinal cord injury.

All participants completed the International Consultation on Incontinence Questionnaire (ICIQ; Avery et al., 2004), Pelvic Floor Distress Inventory Urogenital Distress Inventory (UDI) (Barber et al., 2005), Pelvic Floor Impact Questionnaire (PFIQ) (Barber et al., 2005), and Overactive Bladder Questionnaire (OABq) (Coyne et al., 2002). These are validated questionnaires to assess urinary incontinence symptoms, impact of pelvic floor disorders on daily function, quality of life, symptom bother and health-related quality of life, respectively.

Urine was collected using aseptic technique with a urethral catheter by a trained and licensed practitioner. The total volume of urine was emptied from the participant's bladders. After specimen collection, urine specimens were aliquoted into sterile 50 mL conical tubes and stored at −20°C until further processing. All urine specimens were handled in a sterile biosafety cabinet subsequent to collection.

### DNA extraction

DNA was extracted from microbial pellets formed from the centrifugation of 25–70 mL of urine at 10000 g for 30 min twice. DNA extraction was performed using the cultured cells protocol supplied with the DNeasy Blood and Tissue Kit (Qiagen, Germany). The extracted DNA was quantified and quality checked at A260/A280 nm (Nanodrop, Thermo Fisher Scientific, USA) prior to amplification by polymerase chain reaction (PCR).

### PCR amplification

Bacterial DNA was amplified by PCR using Golay barcoded primers which target the V4 region of 16S rRNA genes (Caporaso et al., 2012). Template DNA was amplified in triplicate using the GoTaq Hot Start Polymerase kit (Promega, USA). One microliter of template DNA and 1 μL of a unique barcoded reverse primer were added to 48 μL of master mix containing 1x colorless reaction buffer, 1.5 mM MgCl_2_, 0.2 mM dNTPs, 0.2 mM forward primer, and 1.25 U of polymerase enzyme. The reaction volumes were placed in a thermocycler and run through the following conditions: 94°C for 3 min (initial denaturation), followed by 35 cycles of 94°C for 45 s (denaturation); 55°C, 40 s (annealing); 72°C, 1.5 min (extension); with a final extension at 72°C for 10 min.

### PCR product purification and sample pooling

Ten microliters of each product was used to verify amplification by gel electrophoresis on a 2% agarose gel. Replicates yielding visible bands at 382 bp were pooled together and purified following the QIAquick PCR Purification kits (Qiagen, Germany) provided protocol. Purified products were again quantified and quality checked at A260/A280 nm (Nanodrop, Thermo Fisher Scientific, USA). Products were diluted to 10 ng/μL and 5 μL of each sample were pooled together for sequencing on the Illumina MiSeq sequencer (Illumina, USA).

### Sequence processing and taxonomic identification

Primers and sequence adapters were removed with the Illumina MiSeq Reporter (version 2.5). The sequences were further processed using scripts implemented through the workflow package Quantitative Insights into Microbial Ecology (QIIME) version 1.9.0 (Caporaso et al., 2010). Individual sequence reads were joined using FASTQ-join (ea-utils, version 1.1.2-537; Aronesty, 2013), with a maximum number of 3 mismatches and minimum overlap of 6. Reads were demultiplexed and filtered with the minimum acceptable Phred score of 21. Reads were checked for alignment to the human genome (assembly GRCh38) with BLAST (version 2.2.22). Operational taxonomic units (OTUs) were identified with a closed-reference approach against the Silva v119 reference database (Quast et al., 2013) using the uSearch (version 5.2.236; Edgar, 2010) algorithm. Chimeric sequences were removed with the blast_fragments approach implemented in identify_chimeric_seqs.py. Taxonomy was assigned to individual OTUs using the RDP Classifier (version 2.2; Wang et al., 2007) with a minimum confidence of 0.80. The resulting OTU table was imported into R for filtering and statistical analysis. Our workflow is provided in the Supplementary Materials (Supplementary Figure 1). The 16S rRNA gene sequences are available for download from the Short Read Archive (SRA) under accession number SUB1600610.

### RT-qPCR to quantify amount of bacterial DNA in urine

Bacterial DNA was amplified by RT-qPCR using the Femto^TM^ Bacterial DNA Quantification Kit (Zymo Research, USA) which contains primers that target the 16S rRNA gene. Two microliters of template DNA, standards, and controls were individually added to 18 μL of Zymos proprietary master mix in triplicate. The reaction volumes were placed in a Chromo4 thermocycler (Bio-Rad Laboratories, USA) and run through the following conditions: 94°C for 10 min (initial denaturation), followed by 40 cycles of 95°C for 30 s (denaturation); 50°C, 30 s (annealing); 72°C, 1 min (extension); with a final extension at 72°C for 7 min. Product amplification and size were verified by running out on a 2% agarose gel at 150 V for 30 min (See Supplementary Figure 2). Amplification data was collected from the thermocycler based on the known input values of the standard curve DNA. This raw data was then used to calculate the total number of bacterial DNA in femtograms present per mL of urine used for DNA extraction.

### Statistical analyses

Differences in baseline characteristics between the two groups (cases and controls) were evaluated using *t*-tests for continuous variables and the Fisher's exact test for categorical variables. All analyses were performed in R (v 3.2.1).

Descriptive statistics and initial visualization of the microbiome data was performed using phyloseq (version 1.14.0, McMurdie and Holmes, 2013). Diversity measures were calculated on the raw count data. Descriptive comparisons of the bacterial communities were performed on relative abundances to account for differences in the number of reads between samples. For these analyses, low abundance OTUs making up < 0.2% of the sample's total number of reads were removed. For stacked barplots and hierarchical clustering, all bacterial genera that had a mean abundance < 0.5% were grouped into an “other” category. Metatstats (White et al., 2009) was used for group comparisons. Hierarchical clustering was performed on the weighted Unifrac distances in R using hclust with the ward.D2 method (Ward, 1963). Pearson correlations were calculated in R using the rcorr function in the Hmisc package (version 3.17-1).

We identified candidate OTUs with differential relative abundance between the UUI and Control urine samples using the DESeq2 package (version 1.8.2; Love et al., 2014) in the R Bioconductor Framework to fit generalized linear models of abundance based on the negative binomial distribution. The significance of the coefficient of the fitted models was inferred with the Wald test. To increase power, we filtered out OTUs with zero counts in >80% of the samples, which reduces the burden of multiple testing correction. Candidate OTUs with an FDR-adjusted *p* < 0.1 were validated as being differentially abundant between groups if the OTU was not driven by a single sample or only present in high-read count samples (samples with total number of reads >300,000), as evaluated by histograms of the raw count data (data not shown).

## Results

All women were between the ages of 42 and 68 years. The women in both groups were similar in BMI, history of pelvic surgery for non-incontinence indications, menopausal status, and estrogen treatment (Table 1). As expected, the UUI group had higher scores on the symptom severity pelvic floor questionnaires (ICIQ, UDI, OABq, *p* < 0.001), increased urgency as measured by the percentage of voids rated with a USS score of 3 or higher (*p* = 0.01) and by average USS (*p* = 0.05), and had lower scores on the OABq Health Related Quality of Life (HRQL *p* = 0.002, see Table 1). The women in the UUI group also had a significant greater number of leaks (*p* = 0.001), but not number of voids per day (*p* = 0.43) or number of nighttime voids (*p* = 0.35) than the control group. All urine specimens were negative for nitrites on a urine dipstick to rule out current UTI.

**Table 1:** **Participant demographics and symptoms**.

	**Control**	**UUI**	***p*-values**
**DEMOGRAPHICS**			
*N*	10	10	ns
Age	58 (9)	57 (8)	ns
BMI (kg/m^2^)	28.3 (6.0)	31.2 (7.8)	ns
Pelvic surgery	5	7	ns
Postmenopausal	7	8	ns
Estrogen treatment	1	1	ns
**SYMPTOM QUESTIONNAIRES**			
International consultation on incontinence questionnaire (ICIQ)	2.0 (1.2)	11.5 (4.8)	< 0.001
Urinary distress inventory (UDI)	0.2 (0.6)	10.4 (5.5)	< 0.001
OABq—symptom severity	6.0 (6.0)	48.3 (20.9)	< 0.001
OABq—Health related quality of life	97.7 (2.5)	66.3 (22.4)	0.002
Percent USS >= 3	10.0 (2.0)	38.5 (23.1)	0.01
Average urgency severity score (USS)	1.6 (0.6)	2.1 (0.6)	0.05
**BLADDER DIARY**			
Urge leaks per day	0 (0)	2.0 (1.3)	0.001
Voids per day	7.4 (2.5)	8.4 (2.2)	ns
Average voids per night	0.2 (0.3)	0.5 (0.8)	ns
**DIET**			
Daily caffeine	7	7	ns
Weekly alcohol consumption	5	3	ns

*Continuous variables are reported as mean (standard deviation), counts reported as the number of individuals with the described demographic. BMI, body mass index; OABq, Overactive Bladder Questionnaire; and USS, Urgency Severity Scale; ns, not significant (based on p < 0.05)*.

Total DNA was extracted from all urine samples in order to quantify bacterial DNA and to characterize the microbial community of the bladder by 16S rRNA gene sequencing. One sample had a low amount of bacterial DNA below the detection limit of the FEMTO quantification kit. This sample was sequenced, but had a low number of reads (~57,000 reads), and was predominantly composed of a known contaminant (Deinococcus-Thermus; Salter et al., 2014). This sample was removed from further analyses, resulting in a 95% success rate for microbiome sequencing and characterization.

**Figure 1:**
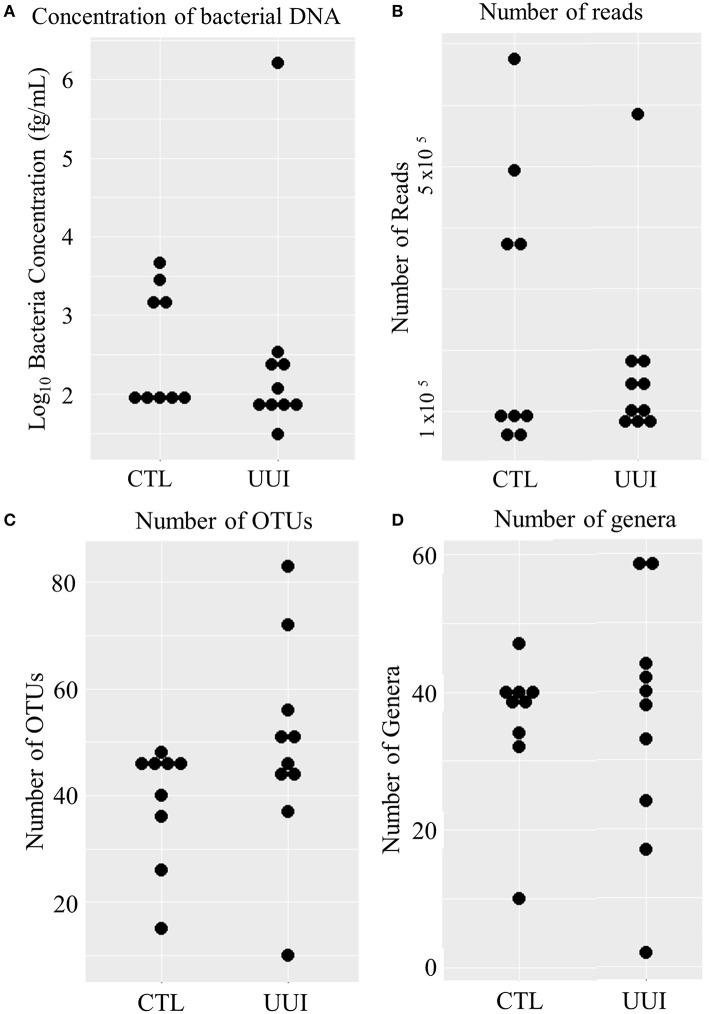
**Distribution of bacteria in urine samples in control and UUI groups**. **(A)** The log transformed concentrations of bacteria. **(B)** The number of reads per sample after preprocessing. These reads were classified into operational taxonomic units **(C)** and further classified into bacterial genera **(D)**. OTUs, operations taxonomic units; CTL, control; UUI, urgency urinary incontinence.

Of the remaining 19 samples (10 cases, 9 controls), the amount of bacterial DNA varied from 31 fg/mL to 1.6 ng/mL (median 95 fg/mL, see Figure 1A), with no difference between cases and controls (median 101 fg/mL in case urine samples, 98 fg/mL control urine samples, *p* = 0.36). After preprocessing and removing non-bacterial and low-count OTUs as described in methods, 3,944,610 reads were classified into 456 OTUs that were used for downstream analyses (see Supplementary Figure 1). The average number of reads classified as bacteria from healthy control (CTL) urine was 252,611 (standard deviation 217,087) and from UUI urine was 167,110 (standard deviation 148,606) and was not significantly different (*p* = 0.34). The large standard deviations are due to five samples (four from control samples and one UUI sample) that had a large number of reads (>300,000) compared to the other samples (range 57,206–190,754 reads per sample, Figure 1B). These samples were flagged to identify if the number of reads skewed any analyses.

### Bacteria in the bladder

**Figure 2:**
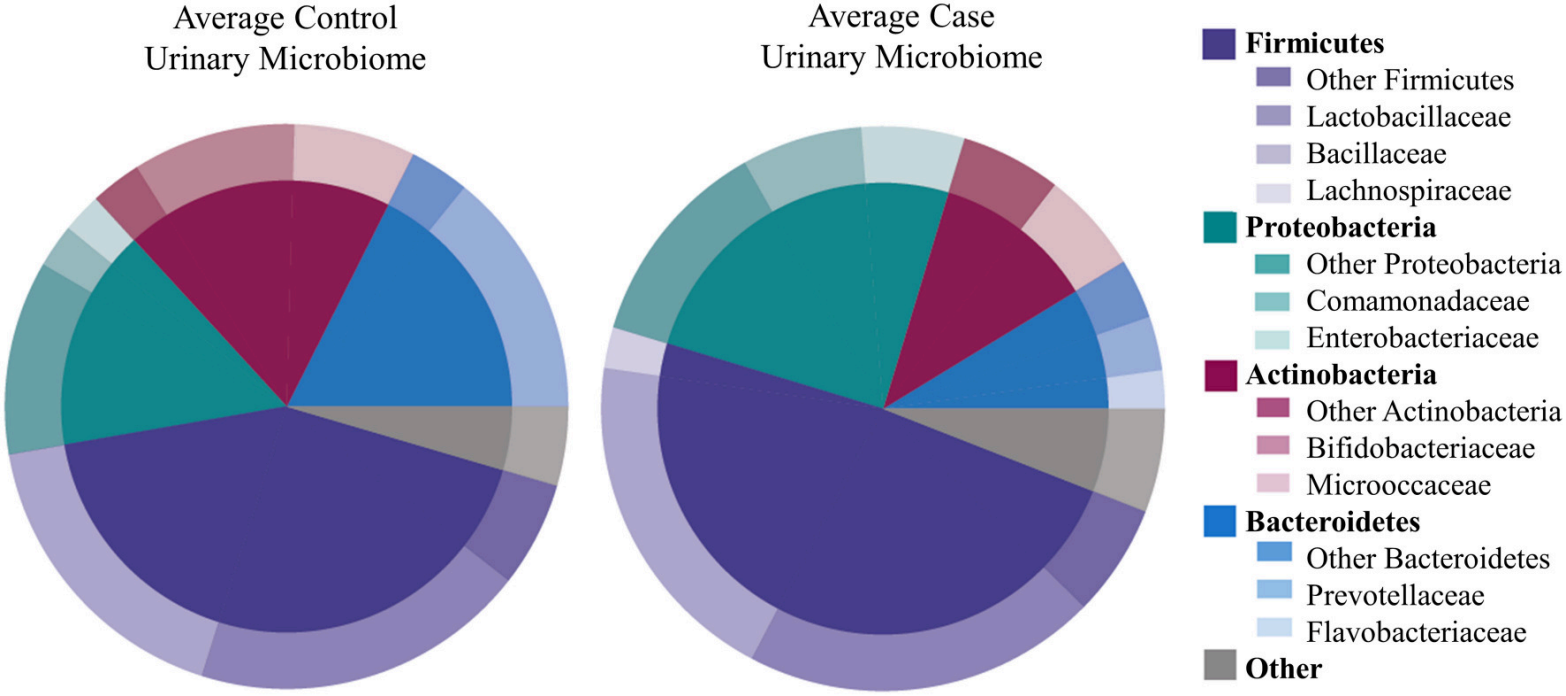
**Microbiome diversity overview between women with normal bladder function (CTL, controls) and women with daily urgency urinary incontinence (UUI, cases)**. At the phyla level (inner circle), the composition is similar with a few slight differences. At the family level (outer circle), however, some differences are apparent such as a marked decrease in Bifodobacteriaceae and Prevotellaceae, and increase in Enterobacteriaceae and Flavobacteriaceae in UUI compared to controls.

We identified 456 unique OTUs, which are considered to be representative of individual bacterial species or a closely related group of species. The average number of OTUs per sample was 39 for controls and 49 for UUI samples (*p* = 0.2, Figure 1C). We identified these OTUs as belonging to 24 different Phyla from all urine specimens. At this level, 97% of the bacteria were classified as either Firmicutes, Proteobacteria, Actinobacteria, or Bacteroidetes. Bacteria from these four phyla were found in most (>17) urine specimens at some level. The average relative abundances for UUI and CTL urine are listed in Table 1 and summarized in Figure 2. Three percent of bacteria were classified as belonging to 16 other Phyla, which were each present only in a subset of the urine samples [between 1 and 14 (5–74%) of the urine samples], typically in low abundance (<16.1%). See Supplementary Table 1 for a list of all bacteria identified from urine specimens.

The most abundantly detected Phylum was Firmicutes (mean OTU frequency: 48.7% UUI, 42.8% CTL), followed by Proteobacteria (mean OTU frequency: 25.0% UUI, 15.9% CTL), Actinobacteria (mean abundance: 11.6% UUI, 19.3% CTL), and Bacteroidetes (mean abundance: 8.7% UUI, 17.6% CTL). The most abundant bacterial families with mean abundances >2% were Bacillaceae, Lactobacillaceae, and Lachnospiraceae (Firmicutes phylum); Prevotellaceae and Flavobacteriacea (Bacteroidetes phylum); Bifidobacteriaceae, and Micrococcaceae (Actinobacteria phylum); Enterobacteriaceae and Comamonadaceae (Proteobacteria phylum, see Table 2, Figure 2). At the genus level, 7 genera had mean abundances >2%. These include Prevotella (Bacteroidetes phylum); Gardnerella and Arthrobacter (Actinobacteria phylum); Escherichia-Shigella (Proteobacteria phylum); and Anoxybacillus, Lactobacillus, and unknown (Firmicutes phylum).

**Table 2:** **Mean relative abundance of bacteria (as percent) in UUI and control urine; reported by Phyla: Family**.

	**Case (%)**	**Control (%)**	***p*-values**
Firmicutes	48.7	42.7	0.60
Bacillaceae	20.3	17.4	0.57
Lactobacillaceae	19.6	19.0	0.98
Lachnospiraceae	2.4	< 2.0	0.69
Proteobacteria	25.0	15.9	0.16
Enterobacteriaceae	6.9	2.5	0.26
Comamonadaceae	5.9	2.3	0.08
Actinobacteria	11.6	19.3	0.40
Micrococcaceae	5.9	7.0	0.60
Bifidobacteriaceae	< 2.0	9.4	0.51
Bacteroidetes	8.4	17.6	0.30
Prevotellaceae	3.1	14.2	0.20
Flavobacteriaceae	2.2	< 2.0	0.35

**Figure 3:**
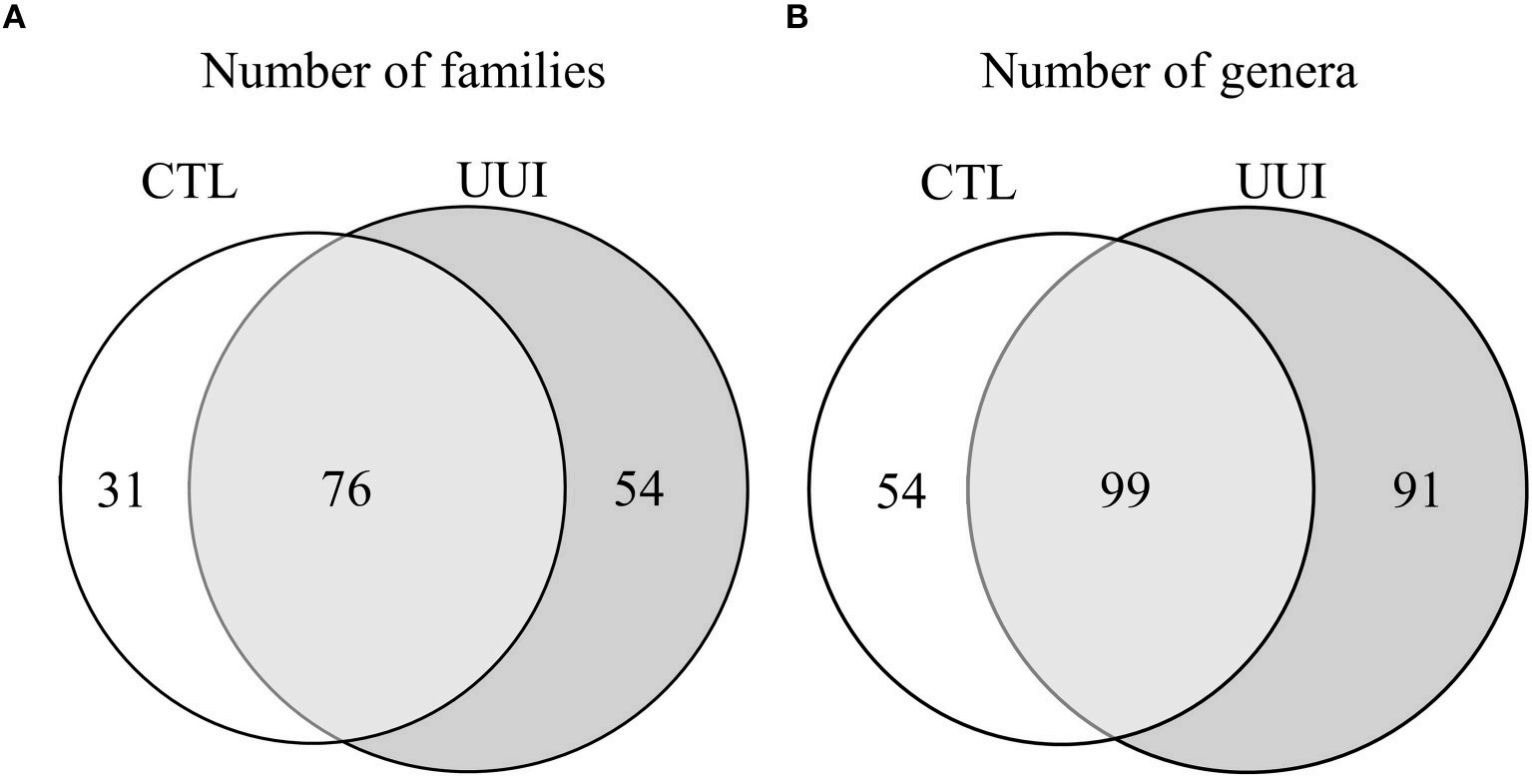
**A Venn diagram depicting the number of bacterial families (A) and genera (B) that are shared and unique between CTL and UUI urine**.

The phyla Firmicutes and Actinobacteria were present in all urine specimens, and Proteobacteria and Bacteroidetes were found in all but one urine specimen. No families were found in all samples. Twenty bacterial families were found in at least half of the samples. Forty-seven percent of bacterial families were identified in both the UUI and CTL urine, 19% families were only identified in control urine, and 34% different families were only identified in UUI urine (Figure 3A). Forty-one percent of the bacterial genera were found in both UUI and CTL urine. Thirty-seven percent genera were only present in UUI urine (4 were present in 3 samples, 8 were present in 2 samples, and the remainder were only present in one sample). Twenty-two percent of genera were only identified in CTL urine (5 were present in two samples, the remainder only present in one sample), see Figure 3B.

### Differentially abundant OTUs

**Figure 4:**
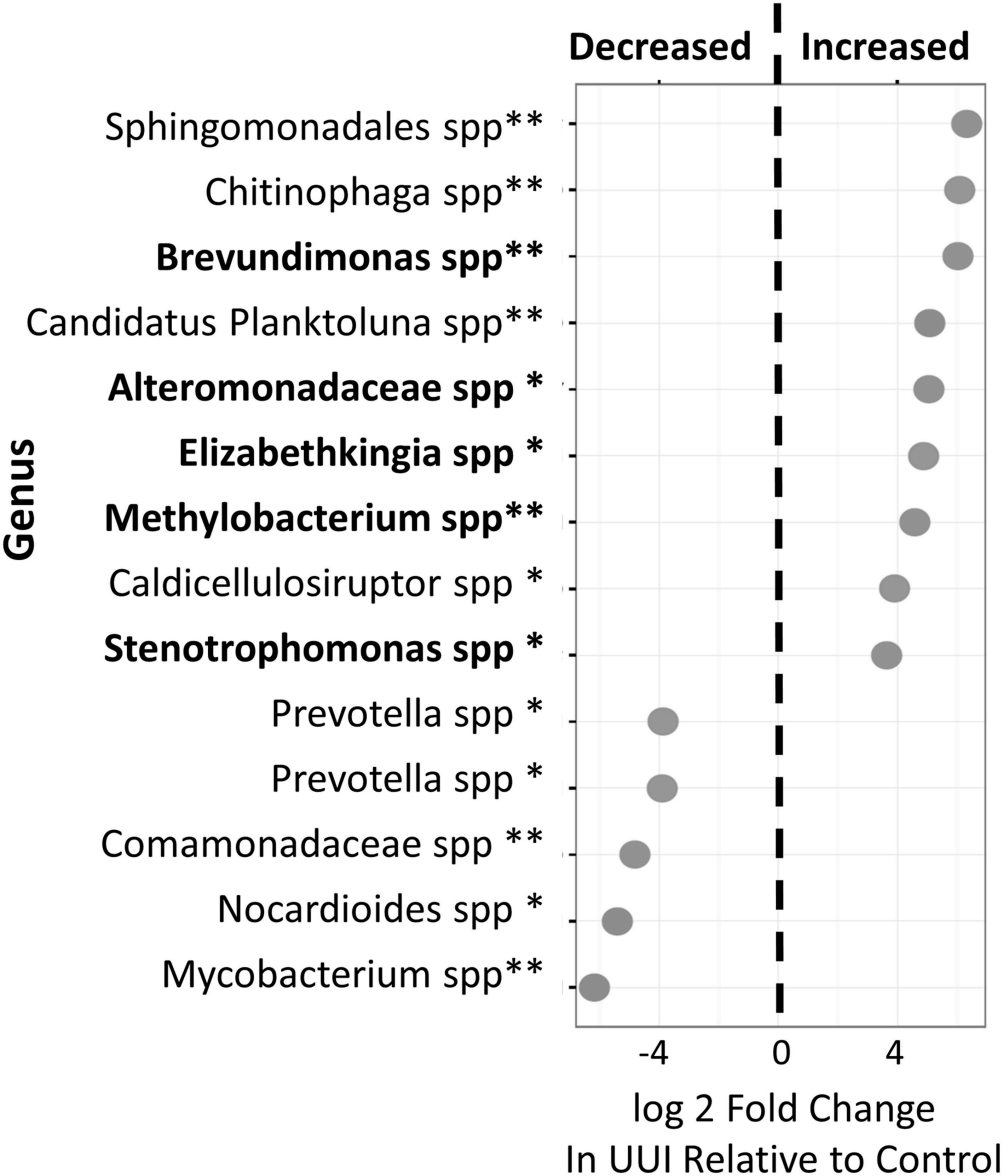
**Differentially abundant bacteria in women with UUI relative to controls**. Many of these bacteria were identified at the genus level. Nine bacteria were increased and five were decreased in women with UUI compared to controls. Of the increased bacteria, five have previously been implicated in UTI (highlighted in bold font). ^*^FDR adjusted *p* < 0.1, ^**^FDR-adjusted *p* < 0.05.

We identified 24 candidate OTUs that are significantly differentially abundant between CTL and UUI groups as described in the methods. Of the candidate OTUs, 14 were verified. Nine OTUs were more abundant and five OTUs were less abundant in UUI compared to controls (Figure 4). Most of the OTUs were classified at the genus level (9), two at the family level and one at the order level. Five of these bacteria (Alteromonadaceae spp.; Krishna et al., 2011), Stenotrophomonas spp. (Moore et al., 1997; Kumar et al., 2015), Brevundimonas spp. (Han and Andrade, 2005), Elizabethkingia spp. (Zong, 2014; Hagiya et al., 2015), and Methylobacterium spp. (Lee et al., 2004) that are increased in UUI have been associated previously with urinary tract infections.

### Individual variability

**Figure 5:**
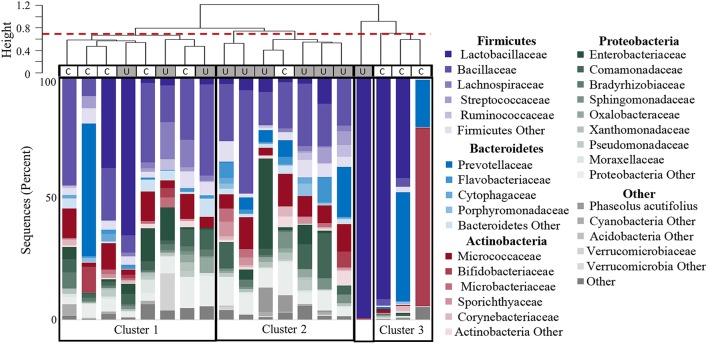
**Clustering of participants based on urinary microbiome profiles**. The dendrogram (upper) is based on hierarchical clustering of the weighted Unifrac distances between the bacteria found in urine samples from controls (C) and women with UUI (U). The dashed line indicates where the clades were divided into three clusters: Cluster 1 which is comprised of five controls and three cases; Cluster 2 which is predominantly cases (six cases, one control), and Cluster 3 which is only controls. The sample type is indicated by the rectangle below the cluster bar: U, case; C, control. One case sample did not fall into a cluster at this level. The stacked bar plots below the heatmap represent the relative abundance of the bacterial families identified by 16S sequences present in each urine specimen. Bacterial families with an overall mean abundance < 0.5%, are grouped as “Other.” The main color is indicative of the bacterial phyla with the shade indicative of bacteria family.

The composition of each individual's urinary microbiome varied greatly, with anywhere from 2 to 49 different bacterial families detected per sample (Figure 5). Four controls and two cases had a microbiota that was dominated by a single bacterial genus (a genus dominating at least 45% of the microbiome sample, as defined by Pearce et al.), while all other samples were diverse. The dominating bacterial genera were Gardnerella (one control), Lactobacillus (one control and two cases), and Prevotella (two controls). The four samples dominated by Lactobacillus and Gardnerella were all from premenopausal women. The number of different bacterial genera in each individual's urinary microbiome ranged from 2 to 59 (cases range 2–59 genera per sample, control range 10–42 genera per sample, Figure 1D).

Hierarchical clustering divided the samples into 4 main groups (Figure 5). The clustering appears to be driven by the diversity of bacterial families. Cluster 1 in Figure 5 is composed of samples with 25–36 different bacterial families (mean 30.9), and contains both CTL and UUI samples (5 CTL and 3 UUI samples). Cluster 2 in Figure 5 contains diverse profiles with 26 to 49 different families (mean 37.3) with no dominating bacterial family, and is composed of 6 UUI and only one CTL sample. One sample does not belong to any clusters and is dominated by one bacterial family. Cluster 3 is composed of three CTL samples that are composed of 9–29 families (mean 14.7).

### Diversity of urinary microbiome

We did not detect any significant differences in alpha diversity measures that are established measures of species richness and distribution within a sample [as measured by the Chao1, Shannon Index, or Inverse Simpson index between control and UUI specimens (Table 3)]. We noticed a great deal of individual variability with the diversity and richness from each sample (Supplementary Figure 3), indicating that the urine microbiome composition not only varies in the type of bacteria that are present but also in the number of different bacteria and abundance of these bacteria.

**Table 3:** **Richness and diversity measures for control and UUI urinary microbiomes**.

	**Controls**				**UUI**				***p*-values**
**Measure**	***Mean***	***SD***	***Min***	***Max***	***Mean***	***SD***	***Min***	***Max***	
Chao1	304.7	77.6	200.2	468.6	304.9	70.1	202.5	423.1	0.995
Shannon	2.6	0.8	1.5	3.5	3.0	1.0	0.5	4.0	0.310
Inverse simpson	8.4	6.9	1.9	22.3	1.1	13.5	1.2	24.3	0.160

*SD, standard deviation; Min, minimum; Max, maximum*.

### Urinary microbiome and clinical symptoms

**Figure 6:**
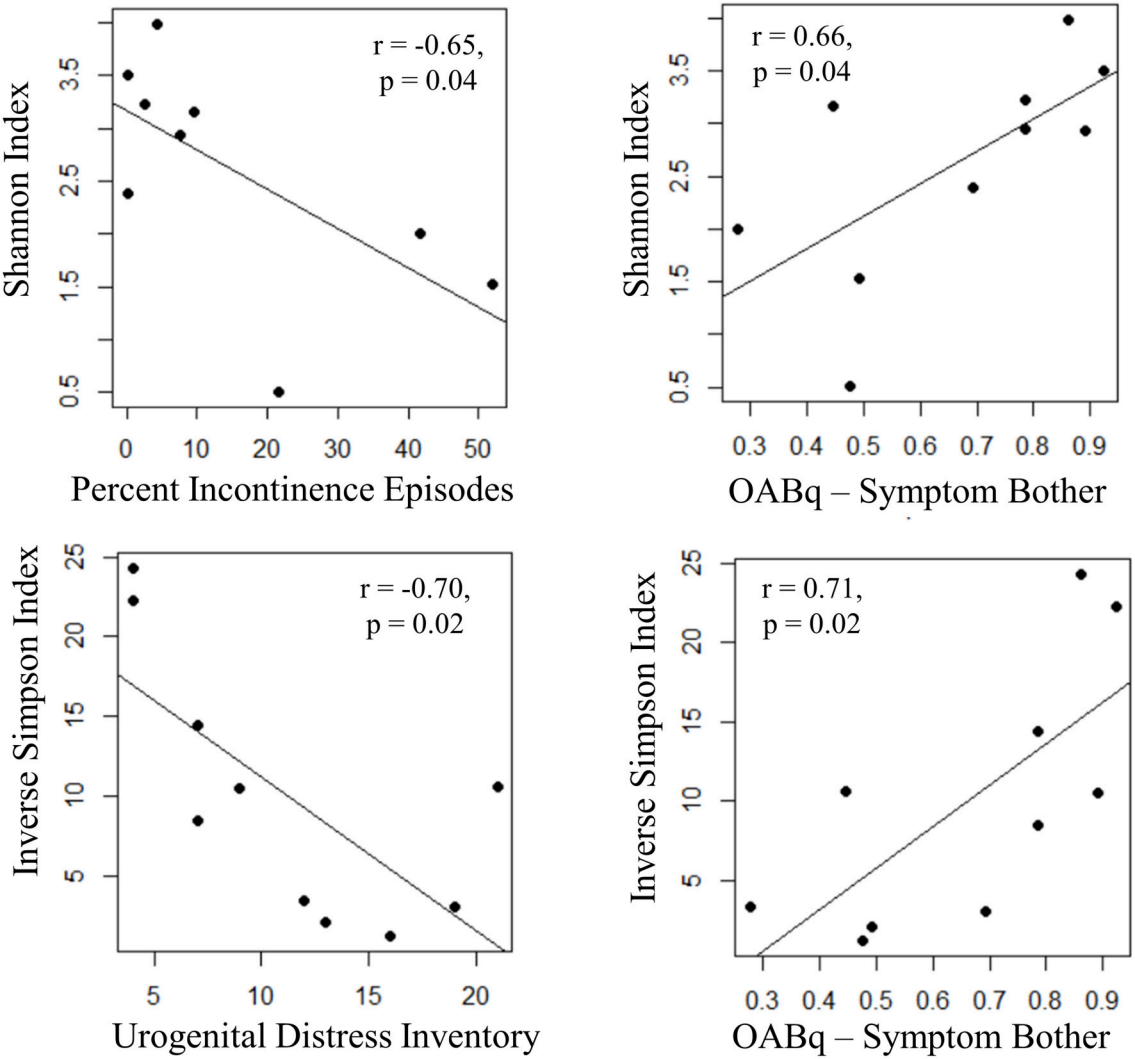
**Increasing UUI symptom severity is associated with decreased microbial diversity in women with UUI**. We identified relationships between the richness and evenness of the urinary microbiome (as measured by the Inverse Simpson and Shannon indices, where lower scores are indicative of lower microbial diversity) and UUI symptom severity. The percent of incontinent episodes is measured by percent of voids with an USS score equal to 4 on a 3 day bladder dairy, and an increase indicates more incontinent episodes. The urogenital distress inventory is a validated questionnaire measuring symptom distress from incontinence and a higher score indicates more distress. Both of these measures have a significant negative correlation with microbial diversity. The OABq-symptom bother is the health related quality of life score from the validated overactive bladder questionnaire. A higher score indicates less symptom bother and is positively correlated with diversity measures. Together, these data indicate that a reduction in microbial diversity is associated with an increase in symptom severity.

Both increasing UDI score (a measure of symptom distress of urinary incontinence) and percent of urgency incontinence episodes (percent of voids where USS = 4) were significantly associated with decreased measures of alpha diversity, as observed for Inverse Simpson index (UDI, *r* = −0.70, *p* = 0.02) and Shannon Index (percent incontinent episodes, *r* = −0.65, *p* = 0.04), respectively. Additionally, the OABq-symptom bother score, where a lower score is indicative of lower quality of life due to OAB symptoms, was positively correlated with both the Shannon and Inverse Simpson indices (Figure 6: *r* = 0.66, *p* = 0.02 and *r* = 0.71, *p* = 0.04, respectively). None of the symptom severity measures where significantly associated with the Chao1 estimator of alpha diversity.

## Discussion

In this prospective pilot study designed to study the urinary microbiome in a clinically well characterized patient population, we have characterized the urinary microbiome of women with normal bladder function and women with UUI using high throughput Illumina MiSeq sequencing of the bacterial 16S rRNA gene. Our results demonstrate important similarities and differences among control and UUI urinary microbiomes.

### Bacteria in urine

Our ability to detect bacteria from almost all of our samples is consistent with the findings of Siddiqui et al. (2011), Siddiqui et al. (2012), and Nelson et al. (2010); but is inconsistent with the findings of Pearce et al. (2014), Pearce et al. (2015), Thomas-White et al. (2016), and Wolfe et al. (2012) who consistently identified bacteria in only about half of the urine specimens collected. This difference could be due to sampling. Our protocol used a minimum volume of 30 mL of urine obtained directly from the bladder through a transurethral catheter, as did the Siddiqui and Nelson protocols. The Pearce et al. and Wolfe et al. protocols used small volumes (1–2 mL) of similarly collected urine. Since urine typically has a low abundance of bacteria, there may be more success with bacterial extraction for amplification from larger volumes of urine as used here. Other relevant considerations may be the high salt content of urine (which may inhibit PCR reactions), presence of large amounts of non-target DNA (e.g., human DNA) and potential contamination by environmental 16S rRNA gene sequences.

Since the exploration of the urinary microbiome is still in its infancy, there are currently no standardized protocols for the collection and extraction of bacterial DNA from urine. Experiments identifying ideal protocols for optimal bacterial extraction would prove valuable to the field, especially for the clinical utility of the urinary microbiome in a clinical setting. Pearce et al's recent work investigating the role of the urinary microbiome in UUI has identified a relationship between the presence of urinary bacteria with a positive response to treatment and decreased risk of UTI (Pearce et al., 2015), and Thomas-White et al has shown a relationship between the urinary microbiota and treatment response (Thomas-White et al., 2016), demonstrating the potential clinical significance of the urinary microbiome. It is important for researchers to consider the sampling methods when interpreting these results and in attempting to validate or replicate these findings. Due to their low sampling volumes, it is possible their “sequence negative” samples are specimens with a low bacterial load which may be sequence positive if a larger volume of urine was used.

### Types of bacteria in urine

Our analysis revealed that human female urine has a polymicrobial composition and that there is substantial variation between urine samples from different individuals. All urine specimens were predominantly composed of bacteria from four phyla: Firmicutes, Bacteroidetes, Proteobacteria, and Actinobacteria, which were also present in most urine specimens to some degree. Firmicutes was the most abundant Phyla, consistent with the findings of Siddiqui and Pearce (Siddiqui et al., 2011, 2012; Pearce et al., 2014, 2015).

Some bacteria present in the urinary microbiome are commonly found in the vaginal microbiome, such as Lactobacillus. These are likely not contamination due to our collection of specimens with a transurethral catheter inserted by an experienced provider using sterile technique. Additional evidence against these being contaminants comes from Wolfe et al. who detected these bacteria from urine specimens collected through suprapubic aspirates (Wolfe et al., 2012).

We noticed a great deal of individual variability with the diversity and richness from each sample, indicating that the urine microbiome composition not only varies in the type of bacteria that are present but also in the number of different bacteria and relative abundance of these bacteria. In our study, only 26% (5 out of 19) samples demonstrated a dominant bacterial genera: one Gardnerella, two Prevotella, and three Lactobacillus. This is different than the findings of the studies by Pearce et al. and Thomas-White (Pearce et al., 2014, 2015; Thomas-White et al., 2016) where they found the majority of their specimens to be dominated by a single genera (>45% of sequences per sample), typically Lactobacillus and Gardnerella. This difference could be due to our small number of participants in our sample, differences in participant populations (our cohort was composed primarily of post-menopausal women that were not using estrogen therapy), differences in urine sample volumes, or preprocessing/filtering techniques used on the data. The three samples that were dominated by the lactic acid producing Lactobacillus were from three out of five pre/perimenopausal women in our cohort. While our sample size is too small for statistical analysis of this finding, it suggests that the urinary microbiome may drastically change after menopause, as is a well-known phenomenon that occurs with the vaginal microbiome (Brotman et al., 2014). These findings suggest that one underlying driver for a shift in urinary microbiome, and hence development of abnormal urinary symptoms and poor urinary control associated with overactive bladder, may be changes in hormonal status associated with menopause.

### The urinary microbiome in UUI

We identified fourteen bacteria with significant differential relative abundances between UUI urine and control urine. Of the nine bacteria that had increased relative abundance in UUI urine, five of these bacteria have been reported as pathogens causing UTI. This suggests that a persistent low grade infection by bacteria that are not commonly detected by routine cultures could potentially be responsible for the irritative symptoms of UUI, at least for some individuals.

We did not detect any differences in the diversity or richness measures between control and UUI specimens. This is consistent with Pearce et al.'s findings, and our diversity estimates were within the range of theirs (Pearce et al., 2014). There was a great deal of individual variability with the diversity and richness from each sample. The high level of individual variability emphasizes the need to look at much larger cohorts to detect group differences.

We also identified significant correlations with symptom severity and alpha diversity measures in women with UUI. Individuals with a lower species diversity had higher scores on the UDI and higher percentage of incontinent episodes. There was also a positive correlation with diversity indices and the OABq-symptom bother questionnaire, where a lower score is indicative of lower quality of life due to OAB symptoms. These data indicate that women with more severe UUI symptoms have a decrease in microbial diversity in their urinary microbiomes.

While our results need to be validated in a larger study, they provide evidence that the diversity of the urinary microbiome may have clinical relevance. Changes in microbial diversity has been linked to disease. In urinary tract disorders, decreased diversity of the urinary microbiome has been linked to in interstitial cystitis (Siddiqui et al., 2012). Urinary microbiome diversity has been associated with response to solifenacin—an orally administered anticholinergic medication used to treat UUI (Thomas-White et al., 2016). Thomas-White found that a lower diversity of cultivatable bacteria was associated with response to low dose of solifenacin and women requiring higher doses or non-responders to have increased diversity of cultivable bacteria, though they did not find this association with the diversity of sequenced microbiota. Decreased microbial diversity of other body sites has also been associated with a variety of clinical conditions such as obesity (Turnbaugh et al., 2009), irritable bowel syndrome (Carroll et al., 2012), and inflammatory bowel disease (Ott et al., 2004).

Pearce et al. also identified bacteria that were associated with UUI (Pearce et al., 2014). They detected an increase in Gardnerella and decrease in Lactobacillus in their UUI population. While we did not detect these differences in Gardnerella or Lactobacillus in our study, an important difference between our study and Pearce et al's study (Pearce et al., 2014) is that our case and control groups were well balanced with respect to menopausal status and estrogen use, where their study had a significantly larger proportion of women in the control group that were “estrogen positive” (premenopausal or on estrogen treatment) than their UUI group. They also detected nine bacteria that were more frequently sequenced or cultured from the UUI cohort using expanded culture techniques. We identified most of these bacteria with an even frequency between controls and cases, and did not detect Oligella, Actinobaculum, or Actinomycyes above our threshold of 0.2% of sequence reads in any of our samples.

## Concluding remarks

These findings have the potential to transform our understanding of the pathogenesis of UUI and other disorders of the bladder. With a larger clinically well-characterized cohort and with the appropriate diagnostic and analytic tools, we may be well positioned to identify a subset of patients with UUI for whom the diversity, presence, or absence of certain bacteria may play a key role in the pathophysiology of their disease. With careful phenotyping of patients and understanding of heterogeneous pathophysiology, it will be possible to identify those patients for whom targeted therapy through restoration of normal urinary microbiome may restore normal bladder function.

## Author contributions

Conceived and designed the experiments: LK, MA, RN. Performed the experiments: PS, SD. Analyzed the data: LK. Wrote the paper: LK. Advised on statistical issues and data analysis: SM. Advised on biological issues/interpretation of data sets: DF, JR, MA, RN, SM, TG. Critical revision of manuscript: DF, JR, MA, RN, SM, TG.

### Conflict of interest statement

The authors declare that the research was conducted in the absence of any commercial or financial relationships that could be construed as a potential conflict of interest. The reviewer DT and handling Editor declared their shared affiliation, and the handling Editor states that the process nevertheless met the standards of a fair and objective review.
