# Supplementary material for: Does the Urinary Microbiome Play a Role in Urgency Urinary Incontinence and Its Severity?
Source: Front Cell Infect Microbiol. 2016 Jul 27;6:78. doi: 10.3389/fcimb.2016.00078 (PMC4961701; doi:10.3389/fcimb.2016.00078)
Supplement: Supplementary file 1 [file DataSheet1.docx]

Supplementary Material

Lisa Karstens, Mark.Asquith, Sean Davin, Patrick Stauffer, Damien Fair, W. Thomas Gregory, James T. Rosenbaum, Shannon McWeeney, Rahel Nardos

Correspondence: Rahel Nardos [nardosr@ohsu.edu](mailto:nardosr@ohsu.edu)

**Supplemental Figure 1.** Our Bioinformatics workflow, with the number of reads remaining at key steps. Python scripts (indicated by .py) are part of the workflow package Quantitative Insights into Microbial Ecology (QIIME) version 1.9.0.

**
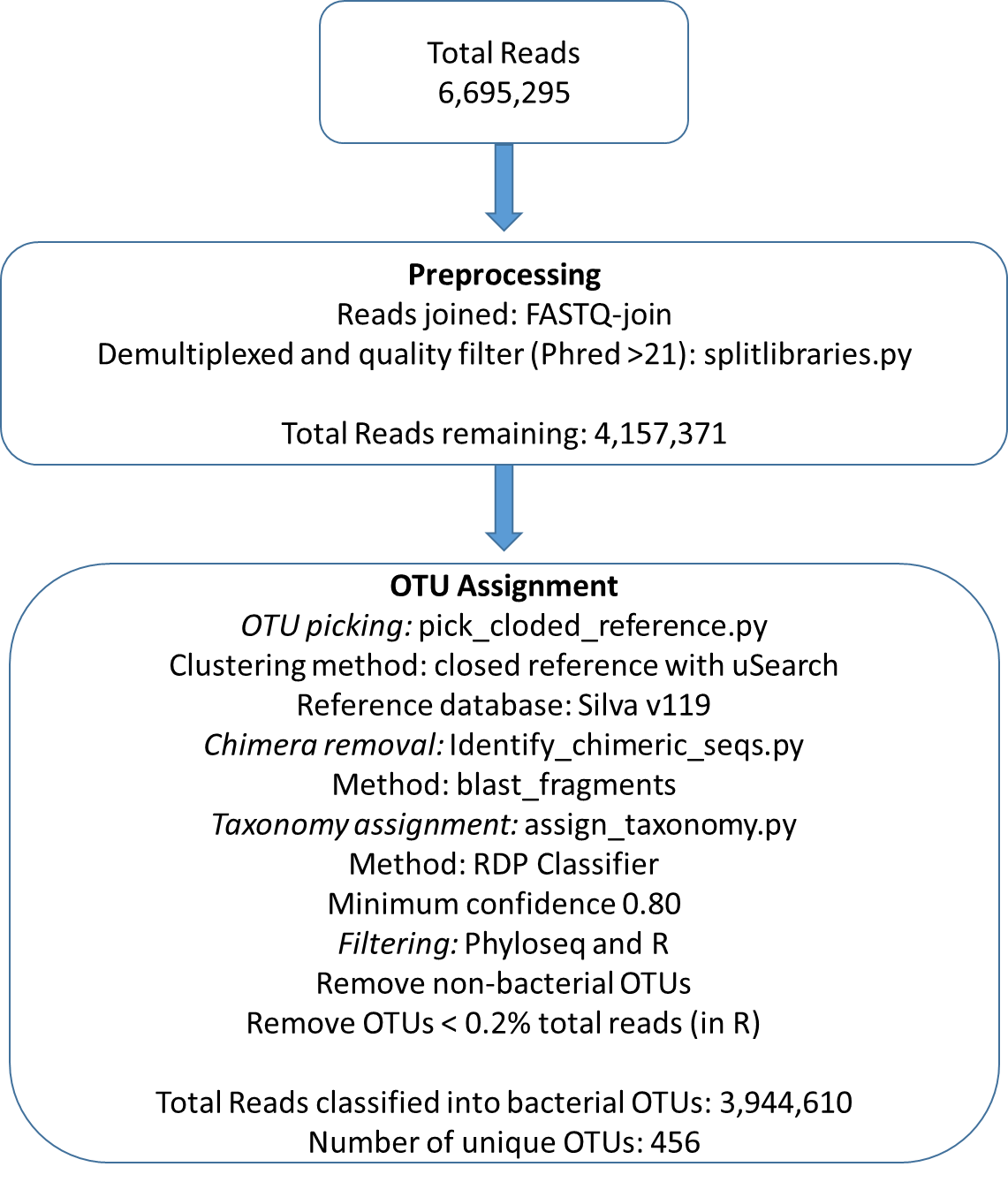
**

**Supplemental Figure 2.** Gels run for product amplification verification. We had an identifiable band in all but one urine specimen (marked with *), and this sample was not used for further analysis. CTL- Urine specimen from a control participant, UUI – Urine from a UUI participant.


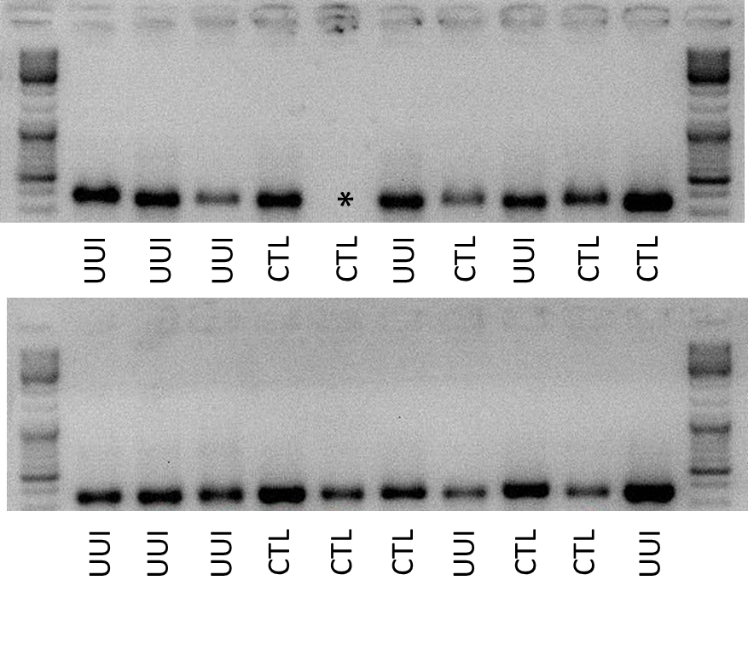


**Supplemental Figure 3.** Alpha diversity of the urinary microbiome. There was a great deal of individual variability with the diversity and richness from each sample. The first through third quartiles of each measure is within the rectangle and the median is indicated by the horizontal line. Outliers are shown as dots not connected to the vertical line.
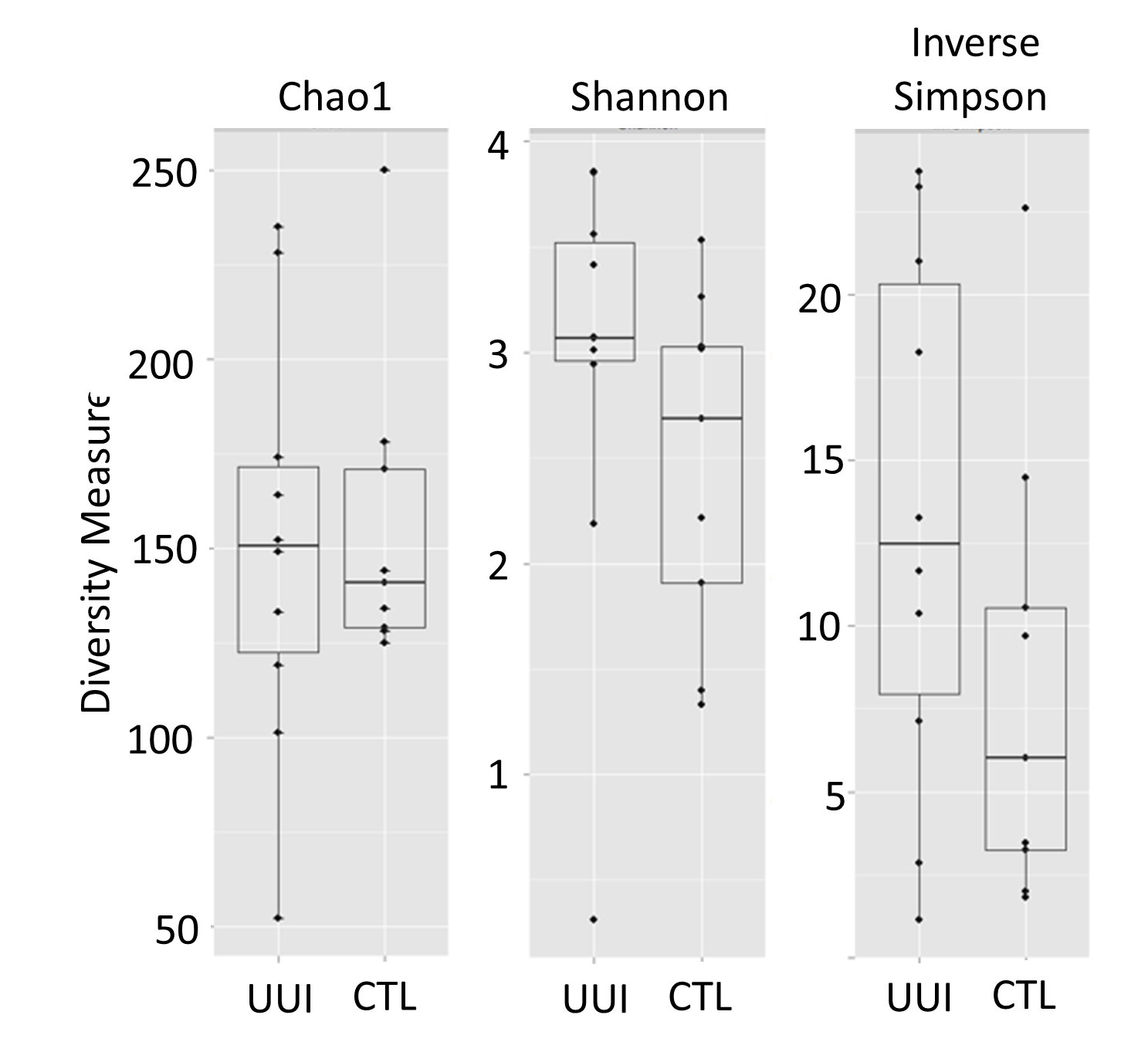


**Supplemental Table 1.** A list of all bacteria Phyla and Genera identified from all urine specimens. Bacteria that were not classified at the genus level are indicated by *unknown* followed by the lowest taxonomic classification identified with 80% confidence by the RDP classifier.

| **Phylum** | | Present in >9 samples | | | | Present in 3 - 9 samples | | | | Present in <3 samples | | | |
| --- | --- | --- | --- | --- | --- | --- | --- | --- | --- | --- | --- | --- | --- |
| **Firmicutes** | |  | | | |  | | | |  | | | |
|  | | *Anoxybacillus* | | | | *Staphylococcus* | | | | *Sporolactobacillus* | | | |
|  | | *unknown Bacillaceae* | | | | *Streptococcus* | | | | *Weissella* | | | |
|  | | *Geobacillus* | | | | *Paenibacillus* | | | | *Clostridium sensu stricto 12* | | | |
|  | | *Lactobacillus* | | | | *Carboxydothermus* | | | | *Anaerococcus* | | | |
|  | | *unknown Bacillales* | | | | *Brevibacillus* | | | | *Parvimonas* | | | |
|  | | *unknown*  *Lachnospiraceae* | | | | *Dialister* | | | | *Incertae Sedis* | | | |
|  | |  | | | | *Bacillus* | | | | *Intestinimonas* | | | |
|  | |  | | | | *Blautia* | | | | *Caldicellulosiruptor* | | | |
|  | |  | | | |  | | | | *Veillonella* | | | |
|  | |  | | | |  | | | | *unknown Bacilli* | | | |
|  | |  | | | |  | | | | *Clostridium sensu stricto 2* | | | |
|  | |  | | | |  | | | | *Finegoldia* | | | |
|  | |  | | | |  | | | | *Acidaminobacter* | | | |
|  | |  | | | |  | | | | *Oribacterium* | | | |
|  | |  | | | |  | | | | *Thermoanaerobacterium* | | | |
|  | |  | | | |  | | | | *Incertae Sedis* | | | |
|  | |  | | | |  | | | | *Aeribacillus* | | | |
|  | |  | | | |  | | | | *Gemella* | | | |
|  | |  | | | |  | | | | *Exiguobacterium* | | | |
|  | |  | | | |  | | | | *unknown Planococcaceae* | | | |
|  | |  | | | |  | | | | *Eremococcus* | | | |
|  | |  | | | |  | | | | *Aerococcus* | | | |
|  | |  | | | |  | | | | *unknown*  *Christensenellaceae* | | | |
|  | |  | | | |  | | | | *Clostridium sensu stricto 1* | | | |
|  | |  | | | |  | | | | *unknown Defluviitaleaceae* | | | |
|  | |  | | | |  | | | | *Shuttleworthia* | | | |
|  | |  | | | |  | | | | *Incertae Sedis* | | | |
|  | |  | | | |  | | | | *Peptostreptococcus* | | | |
|  | |  | | | |  | | | | *Saccharofermentans* | | | |
|  | |  | | | |  | | | | *Fastidiosipila* | | | |
|  | |  | | | |  | | | | *Subdoligranulum* | | | |
|  | |  | | | |  | | | | *Faecalibacterium* | | | |
|  | |  | | | |  | | | | *Anaerotruncus* | | | |
|  | |  | | | |  | | | | *unknown Clostridiales* | | | |
|  | |  | | | |  | | | | *Megasphaera* | | | |
|  | |  | | | |  | | | | *Thermosinus* | | | |
|  | | | | | |  | |  | | | |  |  |
|  | | | | | |  | |  | | | |  |  |
| **Phylum** | | Present in >9 samples | | | | Present in 3 - 9 samples | | | | Present in <3 samples | | | |
| **Proteobacteria** | | | | | |  | |  | | | |  |  |
|  | | *Delftia* | | | | *Acinetobacter* | | | | *Hyphomicrobium* | | | |
|  | | *Bradyrhizobium* | | | | *Brevundimonas* | | | | *unknown*  *Hyphomicrobiaceae* | | | |
|  | | *Escherichia-Shigella* | | | | *Limnohabitans* | | | | *unknown Rhizobiales* | | | |
|  | | *unkown Comamonadaceae* | | | | *Legionella* | | | | *unknown Rhodobacteraceae* | | | |
|  | | *Pseudomonas* | | | | *unknown*  *Sphingomonadales* | | | | *Aegilops tauschii* | | | |
|  | | *Stenotrophomonas* | | | | *Janthinobacterium* | | | | *Undibacterium* | | | |
|  | | *Sphingomonas* | | | | *Tepidiphilus* | | | | *Peredibacter* | | | |
|  | |  | | | | *Campylobacter* | | | | *unknown Enterobacteriaceae* | | | |
|  | |  | | | | *Methylobacterium* | | | | *Haemophilus* | | | |
|  | |  | | | | *Novosphingobium* | | | | *unknown Moraxellaceae* | | | |
|  | |  | | | | *Polynucleobacter* | | | | *Cellvibrio* | | | |
|  | |  | | | | *Pelomonas* | | | | *Silanimonas* | | | |
|  | |  | | | | *unknown Alphaproteobacteria* | | | | *unknown*  *Gammaproteobacteria* | | | |
|  | |  | | | | *Tepidimonas* | | | | *uncultured* | | | |
|  | |  | | | | *Hydrogenophaga* | | | | *unknown Acetobacteraceae* | | | |
|  | |  | | | | *Massilia* | | | | *Candidatus Captivus* | | | |
|  | |  | | | | *Bdellovibrio* | | | | *Altererythrobacter* | | | |
|  | |  | | | | *uncultured*  *Roseobacter sp.* | | | | *unknown*  *Sphingomonadaceae* | | | |
|  | |  | | | | *unknown Myxococcales 0319-6G20* | | | | *Alcaligenes* | | | |
|  | |  | | | | *unknown Alteromonadaceae* | | | | *Hydrogenophilus* | | | |
|  | |  | | | | *Aquicella* | | | | *Methylotenera* | | | |
|  | |  | | | | *unknown Proteobacteria* | | | | *uncultured* | | | |
|  | |  | | | |  | | | | *uncultured prokaryote* | | | |
|  | |  | | | |  | | | | *uncultured Bacteriovorax* | | | |
|  | |  | | | |  | | | | *Arcobacter* | | | |
|  | |  | | | |  | | | | *Coxiella* | | | |
|  | |  | | | |  | | | | *uncultured Proteobacterium* | | | |
|  | |  | | | |  | | | | *Alkanindiges* | | | |
|  | |  | | | |  | | | | *Arenimonas* | | | |
|  | |  | | | |  | | | | *Thermomonas* | | | |
|  | |  | | | |  | | | | *Luteimonas* | | | |
|  | |  | | | |  | | | | *unknown A0839* | | | |
|  | |  | | | |  | | | | *unknown F0723* | | | |
|  | |  | | | |  | | | | *Devosia* | | | |
|  | |  | | | |  | | | | *unknown JG34-KF-361* | | | |
| **Phylum** | | Present in >9 samples | | | | Present in 3 - 9 samples | | | | Present in <3 samples | | | |
|  | |  | | | |  | | | | *Shinella* | | | |
| **Proteobacteria** | | | | | |  | |  | | | |  |  |
|  | |  | | | |  | | | | *Paracoccus* | | | |
|  | |  | | | |  | | | | *Reyranella* | | | |
|  | |  | | | |  | | | | *Caedibacter caryophilus* | | | |
|  | |  | | | |  | | | | *unknown Rickettsiales* | | | |
|  | |  | | | |  | | | | *Rickettsia* | | | |
|  | |  | | | |  | | | | *Sphingobium* | | | |
|  | |  | | | |  | | | | *Sphingopyxis* | | | |
|  | |  | | | |  | | | | *Achromobacter* | | | |
|  | |  | | | |  | | | | *Aquabacterium* | | | |
|  | |  | | | |  | | | | *unknown Oxalobacteraceae* | | | |
|  | |  | | | |  | | | | *unknown Burkholderiales* | | | |
|  | |  | | | |  | | | | *Neisseria* | | | |
|  | |  | | | |  | | | | *unknown Rhodocyclaceae* | | | |
|  | |  | | | |  | | | | *Zoogloea* | | | |
|  | |  | | | |  | | | | *Myxococcales marine metagenome* | | | |
|  | |  | | | |  | | | | *Haliangium* | | | |
|  | |  | | | |  | | | | *Phaselicystis* | | | |
|  | |  | | | |  | | | | *unknown Sandaracinaceae* | | | |
|  | |  | | | |  | | | | *unknown Myxococcales* | | | |
|  | |  | | | |  | | | | *uncultured soil bacterium* | | | |
|  | |  | | | |  | | | | *OM60(NOR5) clade* | | | |
|  | |  | | | |  | | | | *Thiofaba* | | | |
|  | |  | | | |  | | | | *unknown Legionellaceae* | | | |
|  | |  | | | |  | | | | *Unknwon Proteobacteria TA18* | | | |
| **Bacteroidetes** | | | | | |  | |  | | | |  |  |
|  | | *Flavobacterium* | | | | *Prevotella* | | | | *Spirosoma* | | | |
|  | |  | | | | *Porphyromonas* | | | | *unknown Chitinophagaceae* | | | |
|  | |  | | | | *Pseudarcicella* | | | | *Chitinophaga* | | | |
|  | |  | | | | *unknown*  *Sphingobacteriales env.OPS.17 (3)* | | | | *unknown*  *Sphingobacteriales env.OPS.17 (1)* | | | |
|  | |  | | | | *unknown*  *Prevotellaceae* | | | | *Pedobacter* | | | |
|  | |  | | | | *unknown Bacteroidales S24-7* | | | | *Bacteroides* | | | |
|  | |  | | | | *Fluviicola* | | | | *Alloprevotella* | | | |
|  | |  | | | | *Chryseobacterium* | | | | *unknown Cytophagaceae* | | | |
|  | |  | | | | *Elizabethkingia* | | | | *Hymenobacter* | | | |
|  | |  | | | |  | | | | *unknown Sphingobacteriales env.OPS.17 (2)* | | | |
|  | |  | | | |  | | | | *unknown Saprospiraceae* | | | |
| **Phylum** | | Present in >9 samples | | | | Present in 3 - 9 samples | | | | Present in <3 samples | | | |
| **Bacteroidetes** | | | | | |  | |  | | | |  |  |
|  | |  | | | |  | | | | *unknown*  *Sphingobacteriales* | | | |
|  | |  | | | |  | | | | *Proteiniphilum* | | | |
|  | |  | | | |  | | | | *RC9 gut group* | | | |
|  | |  | | | |  | | | | *dgA-11 gut group* | | | |
|  | |  | | | |  | | | | *Blvii28 wastewater-sludge group* | | | |
|  | |  | | | |  | | | | *unknown Bacteroidales S24-7* | | | |
|  | |  | | | |  | | | | *unknown Cytophagaceae* | | | |
|  | |  | | | |  | | | | *Cytophaga* | | | |
|  | |  | | | |  | | | | *Cloacibacterium* | | | |
|  | |  | | | |  | | | | *unknwon Flavobactericeae* | | | |
|  | |  | | | |  | | | | *Hydrotalea* | | | |
|  | |  | | | |  | | | | *Sediminibacterium* | | | |
|  | |  | | | |  | | | | *uncultured bacterium* | | | |
|  | |  | | | |  | | | | *unknown*  *Sphingobacteriaceae* | | | |
| **Actinobacteria** | | | | | |  | |  | | | |  |  |
|  | | *Arthrobacter* | | | | *Corynebacterium* | | | | *Bifidobacterium* | | | |
|  | | *unknown*  *Microbacteriaceae (1)* | | | | *Gardnerella* | | | | *unknown Corynebacteriales* | | | |
|  | |  | | | | *unknown*  *Microbacteriaceae (2)* | | | | *Curtobacterium* | | | |
|  | |  | | | | *Nocardioides* | | | | *Candidatus Planktoluna* | | | |
|  | |  | | | | *unknown Actinobacteria* | | | | *Mobiluncus* | | | |
|  | |  | | | | *unknown Sporichthyaceae* | | | | *Alloscardovia* | | | |
|  | |  | | | | *uncultured* | | | | *Mycobacterium* | | | |
|  | |  | | | | *Sporichthyaceae hgcI clade* | | | | *Dermacoccus* | | | |
|  | |  | | | | *Rubrobacter* | | | | *unknown Acidimicrobiales* | | | |
|  | |  | | | |  | | | | *unknown*  *Geodermatophilaceae* | | | |
|  | |  | | | |  | | | | *Leucobacter* | | | |
|  | |  | | | |  | | | | *unknown PeM15* | | | |
|  | |  | | | |  | | | | *Amycolatopsis* | | | |
|  | |  | | | |  | | | | *unknown Coriobacteriaceae* | | | |
|  | |  | | | |  | | | | *Atopobium* | | | |
|  | |  | | | |  | | | | *unknown*  *Solirubrobacterales 480-2* | | | |
| **Candidate division OD1** | | | |  | | |  | | | |  |  |  |
|  | | *unknown Candidate division OD1 (1)* | | | | *unknown Candidate division OD1 (2)* | | | | *uncultured Parcubacteria bacterium* | | | |
|  | |  | | | |  | | | |  | | | |
| **Phylum** | | Present in >9 samples | | | | Present in 3 - 9 samples | | | | Present in <3 samples | | | |
| **Chloroflexi** |  | | | |  | | | |  | | | |  |
|  | |  | | | | *Chloroflexi bacterium JKG1* | | | |  | | | |
| **Cyanobacteria** | | | | | |  | |  | | | |  |  |
|  | |  | | | | *unknown Chloropast* | | | | *Oryza sativa Japonica Group* | | | |
|  | |  | | | | *Phaseolus acutifolius* | | | | *Virgulinella fragilis* | | | |
|  | |  | | | |  | | | | *Chroococcidiopsis* | | | |
|  | |  | | | |  | | | | *Dacrycarpus imbricatus* | | | |
|  | |  | | | |  | | | | *unknown Gastranaerophilales* | | | |
| **Acidobacteria** | | | | | |  | |  | | | |  |  |
|  | |  | | | |  | | | | *Blastocatella* | | | |
|  | |  | | | |  | | | | *unknown Acidobacteria* | | | |
|  | |  | | | |  | | | | *unknown Acidobacteria* | | | |
|  | |  | | | |  | | | | *unknown Holophagaceae* | | | |
| **Armatimonadetes** | | | |  | | |  | | | |  |  |  |
|  | |  | | | |  | | | | *unknown Armatimonadetes* | | | |
| **BD1-5** | |  | | | |  | | | |  | | | |
|  | |  | | | |  | | | | *unknown BD1-5 (1)* | | | |
|  | |  | | | |  | | | | *unknown BD1-5 (2)* | | | |
|  | |  | | | |  | | | | *unknown BD1-5 (3)* | | | |
|  | |  | | | |  | | | | *uncultured planctomycete* | | | |
| **Candidate division OP11** | | | |  | | |  | | | |  |  |  |
|  | |  | | | |  | | | | *unknown Candidate division*  *OP11* | | | |
| **Candidate division OP3** | | | |  | | |  | | | |  |  |  |
|  | |  | | | |  | | | | *unknown Candidate division*  *OP3* | | | |
| **Candidate division SR1** | | | |  | | |  | | | |  |  |  |
|  | |  | | | |  | | | | *unknown Candidate division*  *SR1* | | | |
| **Candidate division TM7** | | | |  | | |  | | | |  |  |  |
|  | |  | | | |  | | | | *unknown Candidate division*  *TM7* | | | |
| **Elusimicrobia** | | | | | |  | |  | | | |  |  |
|  | |  | | | |  | | | | *unknown Elusimicrobia* | | | |
| **Fusobacteria** | | |  | | |  | | | |  | | | |
|  | |  | | | |  | | | | *Leptotrichia* | | | |
|  | |  | | | |  | | | | *Sneathia* | | | |
|  | |  | | | |  | | | | *unknown Leptotrichiaceae* | | | |
| **Nitrospirae** | |  | | | |  | | | |  | | | |
|  | |  | | | |  | | | | *Nitrospira* | | | |
|  | |  | | | |  | | | |  | | | |
|  | |  | | | |  | | | |  | | | |
|  | |  | | | |  | | | |  | | | |
| **Phylum** | | Present in >9 samples | | | | Present in 3 - 9 samples | | | | Present in <3 samples | | | |
| **Planctomycetes** | | | | | |  | |  | | | |  |  |
|  | |  | | | |  | | | | *SM1A02* | | | |
| **Planctomycetes** | | | | | |  | |  | | | |  |  |
|  | | | | | |  | | *Planctomyces* | | | |  |  |
|  | |  | | | |  | | | | *Gemmata* | | | |
|  | |  | | | |  | | | | *unknown vadinHA49* | | | |
|  | |  | | | |  | | | | *unknown vadinHA49* | | | |
| **SM2F11** | |  | | | |  | | | |  | | | |
|  | |  | | | |  | | | | *unknown SM2F11* | | | |
| **Synergistetes** | | | | | |  | |  | | | |  |  |
|  | |  | | | |  | | | | *Fretibacterium* | | | |
|  | |  | | | |  | | | | *Jonquetella* | | | |
| **Tenericutes** | |  | | | |  | | | |  | | | |
|  | |  | | | |  | | | | *Ureaplasma* | | | |
| **Thermotogae** | | | | | |  | |  | | | |  |  |
|  | |  | | | |  | | | | *Fervidobacterium* | | | |
| **TM6** | |  | | | |  | | | |  | | | |
|  | |  | | | |  | | | | *unknown TM6* | | | |
| **Verrucomicrobia** | | | |  | | |  | | | |  |  |  |
|  | |  | | | |  | | | | *unknown Verrucomicrobia* | | | |
|  | |  | | | |  | | | | *Prosthecobacter* | | | |
|  | |  | | | |  | | | | *Akkermansia* | | | |
